# Older Danes’ Preferences for Their Final Days: A Survey of 1499 Participants

**DOI:** 10.1177/26892820251376358

**Published:** 2025-09-09

**Authors:** Anne Lund Krarup, Matilde A.A. Eriksen, Mike Bundgaard Astorp, Helle Bjørn, Dorte Buchwald, Ove Gaardboe, Dorte Melgaard

**Affiliations:** ^1^ Faculty of Clinical Medicine, Aalborg University, Aalborg, Denmark.; ^2^ Department of Acute Medicine and Trauma Care, EMRUn, Aalborg University Hospital, Aalborg, Denmark.; ^3^ Palliative Care Team, North Denmark Regional Hospital, Hjørring, Denmark.; ^4^ Danish Society for Patient Safety, Frederiksberg, Denmark.

**Keywords:** dying at home, end-of-life care, pain, palliation

## Abstract

**Background:**

Death is inevitable, yet documentation on wishes for the final days of life remains scarce in the literature. Meanwhile, the growing elderly population has brought increased focus on end-of-life care. This study explored wishes and expectations regarding end-of-life care and preferences for the place to die.

**Methods:**

An electronic survey including 12 questions was distributed in May 2024 to approximately 6000 members of the DanAge Association, a Danish nonprofit organization of older adults. The questionnaire was developed in collaboration with health care professionals and DanAge members. Respondents reported gender, age, and postal code but remained otherwise anonymous.

**Results:**

The response rate was approximately 25% with 1499 respondents (72% female, mean age 67 years [standard deviation or SD = 13]). Above half of respondents expressed fear of experiencing pain in their final days (57%), and the majority 87% preferred to be drowsy rather than in pain during this time. Many were also concerned about dying alone (69%) or becoming a practical burden to their loved ones. The preferred place to die was home (61%) followed by hospice (46%). Almost all respondents (94%) wanted to decide on further treatment and their preferred place of death, yet nearly half had not documented their wishes for their final days.

**Conclusions:**

The findings reveal widespread fears about end-of-life experiences, including pain, loneliness, and burdening loved ones. Although most respondents preferred to die at home or in hospice, a significant proportion had not documented their preferences in legal documents. These results highlight the importance of addressing end-of-life concerns and promoting advance directives to align care with individual wishes.

## Background

The global population is experiencing a dramatic demographic shift, with the number of older adults rapidly increasing. In Denmark, it is estimated that by 2053, more than 1 out of 10 will be aged 80 or older.^1^ This trend is mirrored across the rest of the Western world. This demographic transition presents significant challenges for health care systems, particularly in the provision of care for older adults with complex and chronic health conditions. With a growing older population comes increased annual death count. In Denmark, an increase of 10% is expected from 2023 to 2027.^1^ As a result of this demographic development, there is an urgent need to focus on the expansion and improvement of palliative care services. It is essential for health care systems to adapt and ensure that these services are accessible, comprehensive, and adequately resourced.

Meier et al. identified pain relief, maintaining dignity, and emotional well-being as key priorities, while Steinhauser et al. emphasized the importance of alleviating pain, avoiding unnecessary suffering, and respecting patients’ values and preferences.^1,2^ Building on these findings, von Blanckenburg et al. revealed that many terminally ill patients with cancer and their families share fears related to pain, loss of autonomy, and leaving unfinished business, underscoring the universal nature of these concerns.^3^ The fear of pain and the desire for a pain-free state are deeply ingrained concerns, placing the responsibility on clinicians to ensure their patients’ comfort.^4^ A cross-European survey by Bausewein et al. revealed that “burden to others” ranked as the second most common concern for patients with cancer in several countries, following pain. Older age, living arrangements, and prioritizing quality over quantity of life were identified as key factors influencing this concern.^5^ These findings highlight the importance of addressing social and emotional dimensions in palliative care. Advance care planning (ACP) involves discussing and preparing for future medical decisions in the event you become seriously ill or unable to communicate your wishes. The most important part of this process is having meaningful conversations with your loved ones. Many people also choose to document their preferences by completing legal documents known as advance directives.^6^ It is documented that ACP improves end-of-life care and patient and family satisfaction and reduces stress, anxiety, and depression in surviving relatives.^7^ A qualitative study suggests that emotionally driven motives—such as avoiding suffering, maintaining autonomy, staying connected with others, and giving and receiving care—play a significant role in decision making.^8^ In Denmark, ACP can take various forms and occur at different levels. These include “My Last Wishes” (a private document for planning your funeral), a treatment directive, an advance directive or lasting power of attorney, and the option to register in your medical file, for example, that you do not wish to be resuscitated. Additionally, a will focuses on matters that take effect after death, often of a financial nature.

European surveys observed that a mixed population expressed a desire to die at home, surrounded by loved ones, rather than in a hospital setting.^9,10^ In contrast to this, 40% of Danish citizens who died in 2012–2020 died at a hospital.^11^ Few studies examine whether older adults consider it important to write a last will. However, one study reveals that those who are financially better off and have higher levels of education are more likely to create a will.^12^

International studies highlight that a critical aspect of effective palliative care is ensuring that patients experience a death aligned with their values and wishes.^13^ While most research focuses on patients with advanced cancer, only a few studies have explored end-of-life values and preferences within the Danish population.

This study aims to shed light on the expectations surrounding end-of-life care in a Danish population of older adults.

## Methods

On May 2024, an electronic survey was distributed by e-mail to all members (approximately 6000 mail addresses) of DanAge in Aalborg, a part of the national nongovernmental organization for older Danes with 990,000 member. Anyone over the age of 18 can become a member of DanAge. The organization is open to seniors, relatives, and others who wish to support the work for better conditions for the older adults. At the end of May, D.M. (the last author) attended an open-house event, to which all older adults were invited through the daily press, and DanAge members received invitations via e-mail. The event provided an opportunity for those who preferred not to respond electronically to complete a traditional paper questionnaire. Reminders were not sent.

The questionnaire (Supplementary Appendix A1) consists of 12 questions with fixed quantitative response options. The main topics were pain management and comfort in the final days; location, decision making, and end-of-life preferences; and emotional and practical concerns at the end of life. The questionnaire was developed collaboratively by the coauthors, including medical doctors specializing in gastroenterology, acute medicine, and palliative care, as well as a senior researcher in geriatrics. Representatives from DanAge also contributed to its development. The questionnaire was presented to a group of six DanAge members, who expressed how they understood the questions and whether they found them meaningful. Based on their feedback, the questionnaire was revised and reintroduced to the group until consensus was reached that it was clear and meaningful. Most questions were designed to give respondents the possibility to give more than one answer (e.g., “where do you prefer to die” could be answered by both “own home” and “hospice”). Respondents reported their gender, age, and postal code while otherwise remaining anonymous. According to the postal code, respondents were divided into two groups: Group 1, “close to,” includes participants living within 10 kilometers of a hospital, while Group 2 consists of those living more than 10 kilometers away (Supplementary Appendix A2).

The survey was administered via REDCap (Research Electronic Data Capture) tool, where participants were provided with project information and gave their consent by completing the survey.^14,15^ The study was registered and approved as a quality study by the hospital administration, Aalborg University Hospital (ID 2017-011259)

### Statistical analysis

Descriptive statistics were presented with numbers and percentages for categorical variables. Continuous variables were presented as means with standard deviation (SD) or medians and 25th and 75th percentiles (Q1–Q3) for normal and nonnormal data, respectively. Categorical variables were compared using chi-squared tests, and continuous variables were compared using the Wilcoxon rank-sum test or Student’s *t* test as appropriate. Two-sided *p*-values <0.05 indicated statistical significance. All statistical analyses were performed with SAS Enterprise Guide 71 (SAS Institute Inc., Cary, NC, USA).

## Results

The survey was completed by 1499 out of 6000 potential respondents, resulting in a response rate of 25%. As presented in Table 1, the majority of the 1499 participants were women (72%, *N* = 1083). The average age of responders was 67 (SD = 13) years (men 71 [SD = 9.9] years vs. women 65 [SD = 13] years).

**Table 1.** Shows Demographics of Respondents and Their Answers to the Electronic Questionnaire

	All	Hospital distance		Gender	
Subgroup		Close to	Far from	Women	Men
Responders: %, number	100%, 1499	59%, 878	41%, 618	72%, 1083	28%, 413
% Women, number	72%, 1083	69%, 605	77%, 478	See above	See above
Age, mean (standard deviation) years	67 (13)	69 (11)	63 (14)	65 (13)	71 (9.9)
Where do you prefer to die? (more than one answer possible)					
% At home, number	61%, 908	58%, 509^*^	64%, 399	59%, 633^*^	67%, 275
% At a nursing home, number	3.5%, 53	4.7%, 41	1.9%, 12	2.6%, 28^*^	6.1%, 25
% At a hospice, number	46%, 488	47%, 415	44%, 273	51%, 555^**^	32%, 133
% At a hospital, number	3.9%, 59	4.4%, 39	3.2%, 7	3.2%, 35^*^	5.8%, 24
% Other, number	1.9%, 29	1.6%, 14	2.4%, 15	1.7%, 18	2.7%, 11
If my heart stops today, do I want them to attempt resuscitation?					
% Yes, number	61%, 915	59%, 515^*^	65%, 400	62%, 667	60%, 248
% No, number	25%, 377	27%, 234	23%, 143	25%, 271	26%, 106
% Undecided, number	13%, 196	14%, 124	12%, 72	25%, 271	26%, 106
Are you worried about being tormented while you die?					
% Yes, number	57%, 858	58%, 511	56%, 347	60%, 647^**^	51%, 211
% No, number	28%, 425	29%, 252	28%, 173	28%, 302	30%, 123
% Undecided, number	14%, 206	13%, 114	15%, 72	12%, 134^*^	17%, 72
Would you accept to be drowsy when you die if it means avoiding being tormented?					
% Yes, number	87%, 1297	88%, 769	85%, 528	88%, 949	84%, 348
% No, number	2.0%, 30	1.9%, 17	2.1%, 13	1.4%, 15^**^	3.6%, 15
% Undecided, number	11%, 158	9.4%, 83	12%, 75	11%, 115	10%, 43
I want to be able to decide for myself when I die–whether I want more treatment, where I should die, etc.					
% Yes, number	94%, 1406	94%, 825	94%, 581	95%, 1029^*^	91%, 377
% No, number	1.0%, 15	1.1%, 10	0.81%, 5	1.0%, 11	1.0%, 4
% Undecided, number	3.9%, 58	4.0%, 35	3.7%, 23	3.2%, 35^*^	5.6%, 23
Who may fulfil my wishes when I am dying, if I am unable to make decisions?					
% Partner, number	61%, 911	54%, 478^**^	70%, 433	58%, 625^**^	69%, 278
% Children, number	72%, 1080	71%, 620	74%, 460	77%, 831^**^	60%, 249
% Other family members, number	12%, 179	13%, 116	10%, 63	13%, 144^*^	8.5%, 35
% Neighbours/friends, number	1.5%, 22	1.7%, 15	1.1%, 7	1.7%, 18	1.0%, 4
% Health staff, number	7.8%, 117	8.0%, 70	7.6%, 47	8.9%, 96^*^	5.1%, 2
% My general practitioner, number	8.6%, 129	8.3%, 73	9.0%, 56	9.7%, 105^*^	5.8%, 24
% Other, number	1.7%, 26	1.9%, 17	1.5%, 9	1.6%, 17	2.2%, 9
Is there anyone who SHOULD NOT be involved in carrying out my wishes at the end of my life if I am unable to make decisions?					
% Partner, number	3.3%, 50	3.9%, 34	2.6%, 16	3.8%, 41	2.5%, 8
% Children, number	3.1%, 46	2.7%, 24	3.6%, 22	2.4%, 26	4.8%, 20^*^
% Other family members, number	21%, 321	21%, 180	23%, 141	21%, 227	23%, 94
% Neighbours/friends, number	29%, 441	29%, 254	30%, 187	29%, 313	31%, 128
% Health staff, number	12%, 186	11%, 96	15%, 90^*^	12%, 124	15%, 62
% My general practitioner, number	4.7%, 116	7.3%, 64	8.4%, 52	6.9%, 75	9.9%, 41
% Other, number	18%, 275	20%, 173	17%, 102	18%, 197	19%, 78
Dying alone will worry me					
% Yes, number	69%, 1016	71%, 611	67%, 405	73%, 780	58%, 235
% No, number	31%, 455	29%, 253	33%, 202	27%, 287	42%, 168^**^
The thought of becoming a practical burden to my loved ones when I am dying will concern me					
% Yes, number	77%, 1146	77%, 678	76%, 468	76%, 822	79%, 324
% No, number	13%, 187	12%, 104	13%, 83	13%, 172	11%, 45
% Do not know, number	9.2%, 138	9.6%, 84	8.7%, 54	9.6%, 104	8.2%, 34
Have you written down your wishes for when you die?					
% Yes, number in:					
A will	24%, 356	27%, 241	19%, 115^**^	21%, 232	30%, 124^**^
An advance directive (or lasting power of attorney)	31%, 460	33%, 288	28%, 172^*^	31%, 340	29%, 120
A treatment directive (or living will)	6.0%, 90	5.8%, 51	6.3%, 39	6.1%, 66	5.8%, 24
“My last wishes” (a Danish document for planning your funeral)	17%, 248	17%, 145	17%, 103	18%, 191	14%, 57
A health journal	4.1%, 61	3.9%, 34	4.4%, 27	5.1%, 55	1.5%, 6^**^
Other	4.3%, 64	3.5%, 31	5.3%, 33	4.7%, 51	3.2%, 13
% No, number	48%, 713	45%, 395	51%, 318^*^	47%, 510	49%, 203
Have you experienced that a close relative or friend has died in their own home in the past 5 years?					
% Yes, number	30%, 444	28%, 245	32%, 199	30%, 320	31%, 124
How well did you feel your loved one was relieved in the last days?					
% Very good or good, number	68%, 281	66%, 152	71%, 129	66%, 198	72%, 83
% Neither good nor bad, number	11%, 44	12%, 27	9.3%, 17	11%, 33	9.6%, 11
% Very bad or bad, number	8.0%, 33	6.9%, 16	9.3%, 17	9.3%, 28	4.3%, 5
% Do not know, number	14%, 56	16%, 36	11%, 20	13%, 40	14%, 16
Which doctor provided the palliative care?					
% The general practitioner, number	32%, 142	29%, 71	36%, 71	31%, 100	34%, 42
% Out-of-hours medical service, number	6.3%, 28	6.5%, 16	6.0%, 12	7.5%, 24	3.2%, 4
% Hospital doctor, number	8.8%, 39	7.4%, 18	11%, 21	7.8%, 25	11%, 14
% Specialized palliative team, number	32%, 140	31%, 76	32%, 64	33%, 107	27%, 33
% Other doctor, number	2.7%, 12	1.6%, 4	4.0%, 8	2.8%, 9	2.4%, 3
% Do not know, number	23%, 102	27%, 66	18%, 36^*^	22%, 71	25%, 31

^*^ *p* < 0.05 within subgroup.

^**^ *p* < 0.001 within subgroup.

### Overall preferences among respondents

**FIG. 1.**
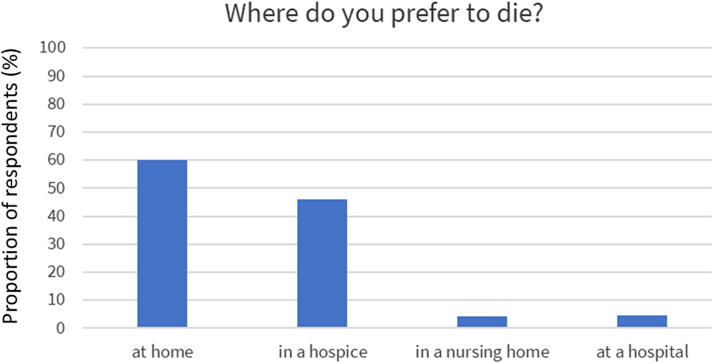
Preference of place to die among 1499 DanAge members.

**FIG. 2.**
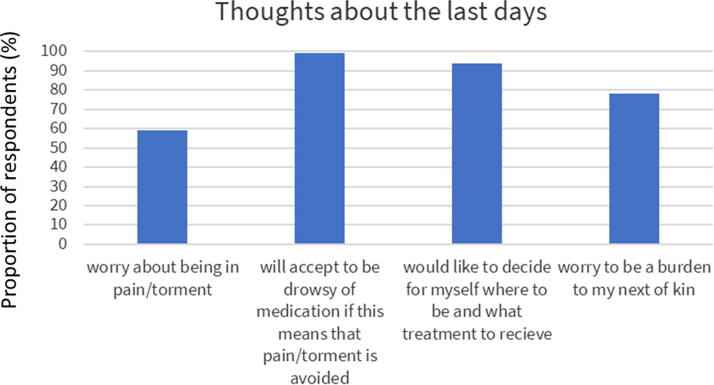
Thoughts about the last days of life among 1499 DanAge members.

**FIG. 3.**
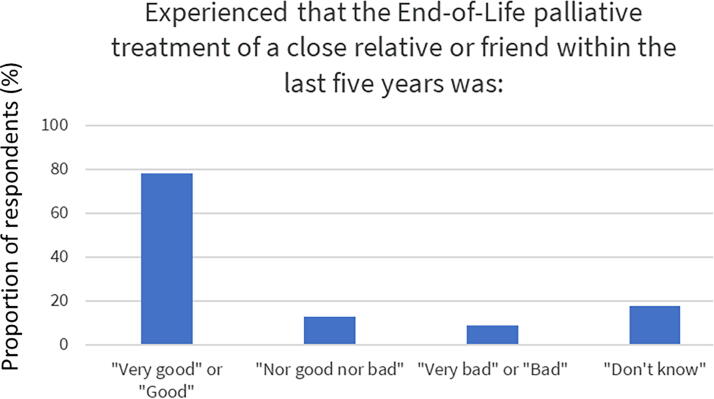
Experience of end-of-life palliative treatment for a close relative or friend within the last five years.

Being able to decide what treatment to receive and where to be located when ending life was preferred by almost all (94%). However, only half of the responders had written down their wishes (48%). The preferred places to die were at home (61%) or hospice (46%), whereas less popular places were hospitals (3.9%) or nursing homes (3.5%), Figure 1. Most respondents were concerned about being tormented by symptoms at the end of life (57%, Table 1, Fig. 2). This may be well connected to the observation that the vast majority would rather be drowsy due to medication than having symptoms (87%, Table 1, Fig. 2). Respondents’ previous experience with end-of-life care among close relatives can be seen in Figure 3.

### Difference between women and men

The responses were quite similar between genders. However, more women than men wanted to die in a hospice (51% of women vs. 32% of men, *p*-value <0.001). Moreover, the thought of dying alone was more often worrisome for women than men (73% vs. 58% of men, *p*-value <0.001). Also, more women were concerned about being tormented by symptoms (60% vs. 51% of men, *p*-value 0.003). If unable to make decisions on their own, when dying, more women reported that decisions should be made by their children (77% vs. 60% of men, *p*-value <0.0001). Men were, on the contrary, more likely to report their partner as the one responsible for decision making (69% vs. 58% of women, *p* < 0.0001). They were also more likely to have had a will made (30% vs. 21% of women, Table 1).

### Living close to or far from a hospital influenced some answers

Surprisingly, people living far from a hospital were more prone to wish a resuscitation attempt should they experience a cardiac arrest (59% living close to vs. 65% living further away, *p*-value = 0.02). The people living far from a hospital relied more on their partner if unable to make decisions (70% vs. 54%, *p*-value <0.0001). They also were less likely to have written down their wishes in, for example, a will compared with those living closer to a hospital (51% vs. 45%, *p*-value = 0.02).

## Discussion

The findings in this survey highlight common fears about end of life, including pain, loneliness, and burdening loved ones. While most preferred to die at home or in hospice, many had not documented their wishes. A review also shows that many older adults have limited knowledge of ACP, although they are generally positive about having a say in their final stage of life.^16^

A substantial proportion of respondents (57%) expressed concerns about experiencing symptoms at the end of life, which aligns with Meier’s findings that pain relief, maintaining dignity, and emotional well-being are key priorities.^2^ Additionally, 87% indicated they would prefer to be drowsy from medication rather than suffer from symptoms. These preferences reflect universal concerns identified by Steinhauser et al., who emphasized the importance of alleviating pain, avoiding unnecessary suffering, and respecting patient values and preferences.^1^ Similarly, von Blanckenburg et al. noted that fears related to pain, loss of autonomy, and unfinished business are common among terminally ill patients and their families.^3^ According to Danish legislation, a terminally ill patient may receive the pain-relieving, sedative, or similar medication necessary to alleviate their condition, even if this may result in the hastening of death.

The fear of becoming a burden was also a prominent concern, with a cross-European survey by Bausewein et al. identifying “burden to others” as the second most common concern after pain.^5^

European studies have consistently found that many patients wish to die at home, surrounded by loved ones, rather than in a hospital setting.^9,10,17^ This aligns with the findings of the current study, where home was the most preferred location. But it does not fit well with the Danish reality where >40% die at hospital.^11^ This has been achieved also for patients who are chronically ill but experience acute deterioration during out-of-office hours. Few studies have examined older adults’ perspectives on writing a last will, but encouraging discussions and providing support for documenting end-of-life wishes could help bridge the gap between preferences and actions.

Gender differences in end-of-life preferences were notable in the current study. More women than men expressed a preference for dying in a hospice and were more concerned about dying alone. Women also reported greater fear of being tormented by symptoms. When unable to make decisions themselves, women were more likely to designate their children as decision-makers, while men were more likely to rely on their partner. Additionally, men were more likely to have prepared a will. These findings align with Skulason et al., who identified gender differences in discussing one’s own impending death. Their study revealed that women are more likely to express concerns about emotional and relational aspects of dying, whereas men tend to focus on practical matters such as documentation and decision making.^18^ These gendered differences in end-of-life priorities and fears highlight the need for tailored approaches in palliative care to address diverse concerns effectively.

Living in proximity to health care facilities also influenced end-of-life preferences. Respondents living farther from hospitals were more likely to wish for resuscitation attempts in the event of cardiac arrest and tended to rely more on their partner for decision making if they became unable to decide for themselves. However, these individuals were less likely to have documented their end-of-life wishes, such as through a will, compared with those living closer to hospitals. This aligns with findings by Downing and Jack, who highlighted unique challenges in rural end-of-life care, including reduced access to health care services and reliance on close family for support.^19^ These disparities emphasize the importance of addressing geographic barriers in palliative care, ensuring that rural populations receive equitable access to resources for documenting and honoring their preferences. It was, however, a surprise that respondents living in rural areas were more likely to want a resuscitation attempt if dying. We had the expectation that rural citizens were more accepting of life and death and would be more likely to just accept that when the heart stops, you die. This is the typical way of seeing life at the west and north coast of Denmark and fits well with the terms of life in these rugged countrysides. However, the rural areas in this study were still close to a hospital (<50 km), which might have influenced results.

A strength of this survey is that, despite the questions not being standardized or validated, they were developed in close collaboration between members of the DanAge Association and a multidisciplinary group of health care professionals. The questionnaire was reviewed and commented on by several members of the DanAge Association. Similarly, the setup in REDCap was thoroughly tested by the members to ensure it was intuitive. To accommodate those who preferred not to respond online, D.M. (the last author) was present at an open-house event where participants could complete the questionnaire in person. A limitation is that the respondent group is likely dominated by healthy older adults, and with a minor group of relatively younger participants, making it difficult to predict whether their answers would differ if their daily lives were impacted by illness and reduced functional ability. The response rate is 25%, which is a limitation of the study.

## Conclusion

The findings from this survey highlight prevalent concerns about end-of-life experiences, with many respondents expressing concerns about pain, loneliness, and the potential burden their death may place on loved ones. Despite these anxieties, the majority of respondents expressed a preference to die at home or in a hospice, rather than in a hospital or nursing home—settings where most people currently pass away.

A significant number had not formally documented their end-of-life wishes, highlighting a critical gap in ACP. This suggests that many individuals may not have created an advance directive or engaged in discussions about their broader care preferences. The results emphasize the need for open conversations about where and how individuals wish to spend their final days, as well as initiatives to encourage advance planning. Such actions can help alleviate fears, empower individuals, and ultimately improve the quality of end-of-life care.

## Notes

Supplementary Appendix A1

Supplementary Appendix A2

## Abbreviations Usd

ACP: Advance Care planning
REDCap: Research Electronic Data Capture
