# Supplementary material for: Older Danes’ Preferences for Their Final Days: A Survey of 1499 Participants
Source: Palliat Med Rep. 2025 Sep 9;6(1):424–31. doi: 10.1177/26892820251376358 (PMC12528844; doi:10.1177/26892820251376358)
Supplement: Supplementary Appendix A1 [file 26892820251376358_supplementary_appendix_a1.docx]

Questionnaire

|  | Question |  |
| --- | --- | --- |
| 1 | Gender | 🞎 Male  🞎 Female |
| 2 | Age |  |
| 3 | Postcode |  |
| 4 | Are you worried about being in pain in the final period? | 🞎 Yes  🞎 No |
| 5 | Would you accept being drowsy in the final days if it meant avoiding pain? | 🞎 Yes  🞎 No |
| 6* | Where would you prefer to be in the final period? | 🞎 At home  🞎 Care home  🞎 Hospice  🞎 Hospital  🞎 Other |
| 7 | I want to decide how my final days should be – whether I want more treatment, where I should die, etc. | 🞎 Yes  🞎 No |
| 8* | Who should carry out my wishes in the final period if I am unable to make decisions? | 🞎 Partner  🞎 Children  🞎 Neighbour/friends  🞎 Care staff  🞎 Own doctor  🞎 Other |
| 9 | If my heart stopped today, would I want resuscitation efforts? | 🞎 Yes  🞎 No |
| 10 | Dying alone would worry me | 🞎 Yes  🞎 No |
| 11 | Being a practical burden on my loved ones in the final period would worry me | 🞎 Yes  🞎 No |
| 12* | Have you written down your wishes for the final period? | 🞎 Will  🞎 Lasting power of attorney  🞎 Advanced directive  🞎 My final wishes  🞎 Health record  🞎 Other |

* Multiple responses allowed
