# Supplementary figures and images for: Older Danes’ Preferences for Their Final Days: A Survey of 1499 Participants
Source: Palliat Med Rep. 2025 Sep 9;6(1):424–31. doi: 10.1177/26892820251376358 (PMC12528844; doi:10.1177/26892820251376358)

Map


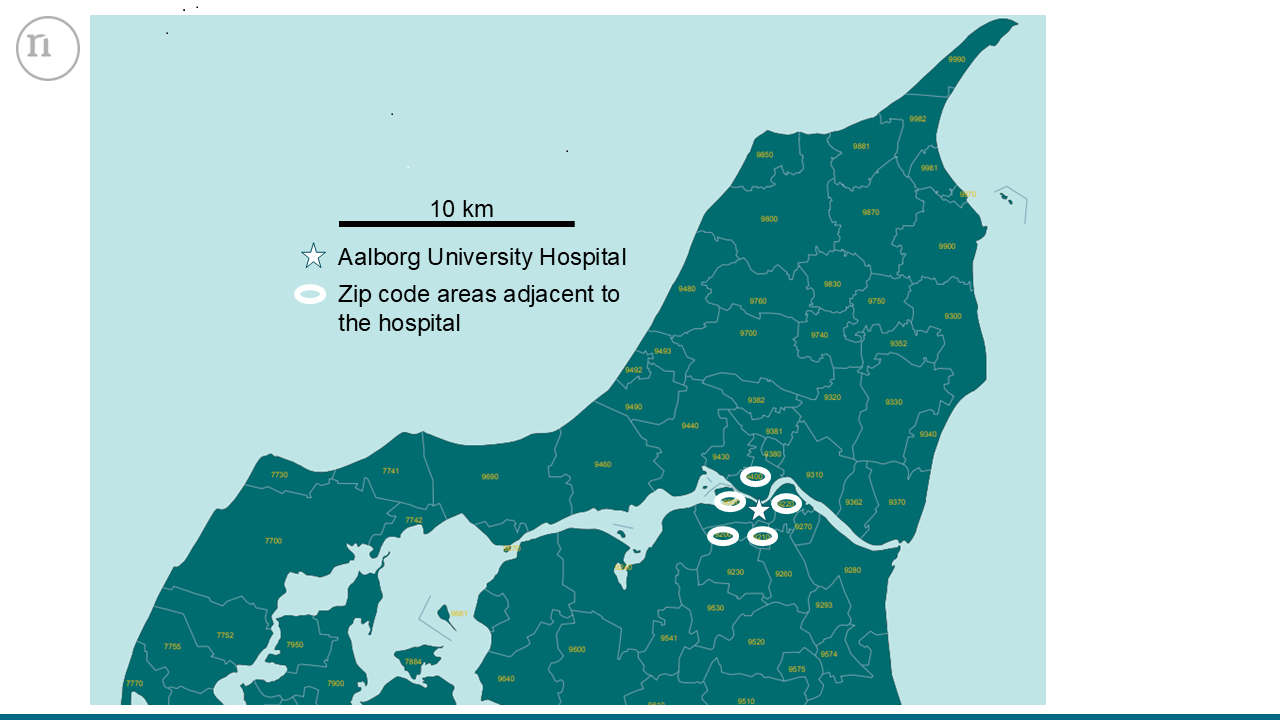

Supplement: Supplementary Appendix A2 [file 26892820251376358_supplementary_appendix_a2.docx]
